# Molecular epidemiology of *Theileria equi* in horses and their association with possible tick vectors in the state of Rio de Janeiro, Brazil

**DOI:** 10.1007/s00436-013-3360-0

**Published:** 2013-03-09

**Authors:** Maristela Peckle, Marcus S. Pires, Tiago M. dos Santos, Erica C. R. Roier, Claudia B. da Silva, Joice A. R. Vilela, Huarrisson A. Santos, Carlos L. Massard

**Affiliations:** 1 Animal Parasitology Department, Veterinary Institute, Universidade Federal Rural do Rio de Janeiro, BR 465, Km 7, Seropédica, Rio de Janeiro, 23897-000 Brazil; 2 Zootechny Department, Agricultural Science and Technology Institute, Universidade Federal de Mato Grosso, Rondonópolis, Brazil; 3 Epidemiology and Public Health Department, Veterinary Institute, Universidade Federal Rural do Rio de Janeiro, Seropédica, Brazil

## Abstract

The aim of this study was to detect *Theileria equi* (Laveran 1901) DNA in horses and ticks using real-time PCR and to list the factors associated with infection in animals located in the Seropedica and Petropolis municipalities of the state of Rio de Janeiro. We tested blood samples from 314 horses and samples from 300 ticks, including 191 *Amblyomma cajennense*, 104 *Dermacentor nitens*, and 5 Ixodida larvae. Factors inherent to the horse, the ownership, and animal management were obtained from an epidemiological questionnaire and were evaluated in association with the presence of *T*. *equi* DNA in the animals. Among the horses in the study, 81 % (*n* = 253/314) presented *T*. *equi* DNA, and the animals of the Seropedica municipality had the highest infection frequency (91 %, *n* = 128/141, *p* < 0.001). The factors that had significantly different infection frequencies by chi-squared or Fisher’s exact tests (*p* < 0.2) were included in a logistic regression model using the R programming package. Work and walking activity (odds ratio [OR] = 5.7, CI = 2.3–14.4), reproductive activity (OR = 3.8, CI = 1.3–11.5), and tick infestation (OR = 2.6, CI = 1.1–6.2) were factors that favored the presence of *T*. *equi* DNA in the animals (*p* < 0.05). Among the tick samples, *A*. *cajennense* and *D*. *nitens* were the identified species. The presence of *T*. *equi* DNA was observed in 9.9 % (*n* = 19/191) of the *A*. *cajennense* samples and 3.8 % (*n* = 4/104) of the *D*. *nitens* samples. A multivariate analysis revealed that the presence of *A*. *cajennense* on the animals (OR = 4.1, CI = 1.8–9.1) was associated with the presence of *T*. *equi* DNA in the horses. In the studied municipalities, activities related to work, walking, and reproduction and the presence of ticks on the horses, particularly an intense infestation of *A*. *cajennense*, are factors that lead to infection with *T*. *equi* in the horses.

## Introduction

Equine theileriosis is a disease caused by an intra-erythrocytic protozoan *Theileria equi* (Laveran 1901) (Mehlhorn and Schein 1998) and is characterized by fever, anemia, jaundice, hepatomegaly, splenomegaly, and hemoglobinuria. However, most infected animals became asymptomatic carriers, acting as a source of infection for tick vectors (De Waal 1992). In these animals, a low parasitemia level often precludes microscopic diagnosis by Giemsa-stained blood smears (Böse et al. 1995).

The worldwide problem of equine piroplasmosis is considered the main impediment to the international movement of horses because positive or seropositive animals are denied either entry for sports competitions or final export into several countries considered free of the disease (Knowles 1996; Friedhoff 1990).

The biological agent transmission occurs via the inoculation of sporozoites in horses by a tick vector. To date, ten tick species from three distinct genera (*Dermacentor*, *Rhipicephalus*, and *Hyalomma*) have been identified in *T*. *equi* transmission (Mehlhorn and Schein 1998). Until now in the Americas, *Dermacentor variabilis* and *Rhipicephalus microplus* (Canestrini 1887) have been tick species identified as transmitters of *T*. *equi* in experimental conditions (Stiller and Coan 1995; Guimarães et al. 1998a; Ueti et al. 2005; Ueti et al. 2008). In Latin America, horses are regularly infested with three species of ticks: *Dermacentor nitens*, *Rhipicephalus microplus*, and *Amblyomma cajennense* (Borges and Leite 1998; Labruna et al. 2001; Da Costa Pereira et al. 2005). *R*. *microplus* has been implicated as a vector (Guimarães et al. 1998a, b; Ueti et al. 2005), but it is a monoxenic tick, and *T*. *equi* transovarial transmission has not yet been reported. Thus, its epidemiological importance has been questioned. The role of *D*. *nitens* in the transmission of *T*. *equi* has not been reported (Denning 1988). In addition to biological transmission, congenital and iatrogenic forms of transmission have been observed (Tenter and Friedhoff 1986; Allsopp et al. 2007; Santos et al. 2008).

*Theileria equi* remains in the infected animal body for years and possibly throughout the animal’s life (Mehlhorn and Schein 1998). Thus, the real-time polymerase chain reaction (qPCR), which is highly sensitive and specific, is a molecular technique that can be used to quantify the actual circulation of the agent in the studied area, in addition to allowing the quantification and investigation of circulating strains. Few studies have been conducted using molecular tools to investigate the epidemiological aspects of equine theileriosis in Brazil.

This study aimed to detect *T*. *equi* in horses and ticks by qPCR and to relate the variables about the host, environment and potential tick vectors to the presence of *T*. *equi* DNA in horses domiciled in the Seropedica and Petropolis municipalities of the state of Rio de Janeiro, Brazil.

## Materials and methods

### Sampling

From January to May 2009, 30 properties with horses in the Seropedica and Petropolis municipalities of the state of Rio de Janeiro, Brazil, were analyzed. The sampling was calculated according to an equation described by Sampaio (2002), and the collection was performed by convenience in the studied area, with an assumed expected frequency of 59 % (Heim et al. 2007), an error of 5 % and an accuracy of 9 %. Blood samples were collected from 314 horses (*n* = 173 in Petropolis and *n* = 141 in Seropedica), and 300 samples were collected from ticks on these horses.

After the horse owners consented to the procedure, the animals were inspected for the presence of ticks, and the infestation level was assessed, followed by the collection of ticks. The blood samples were collected by jugular venipuncture and placed under vacuum in sterile tubes containing ethylenediamine tetraacetic acid (EDTA) anticoagulant.

An average of ten tick specimens in various developmental stages was collected from the different body parts of each infested animal. The taxonomic identification was based on the dichotomous keys of Aragão and Fonseca (1961) and Barros-Battesti et al. (2006). After identification, these specimens were stored in polypropylene tubes containing isopropyl alcohol according to the species, developmental stage, and sexual dimorphism. The infestation level of the ticks on the horses was measured subjectively based on the observation of adult tick specimens on the horses, categorized, and sorted into two infestation levels (absent/low or moderate/high) according to Labruna et al. (2001).

### Epidemiological questionnaire

To assess the possible factors associated with infection by *T*. *equi* in the horses, a semi-structured questionnaire about the horses and the breeding and property management characteristics was administered to the owners. The factors investigated by the questionnaire were organized as follows: municipalities of Seropedica (low-altitude regions, less than 400 m in elevation) or Petropolis (high-altitude regions, more than 400 m in elevation); satisfactory or unsatisfactory zootechnical and sanitary management (Santos et al. 2011); presence or absence of ticks on animals; horses bred in close contact with cattle (cattle and horses shared the same pasture or were located close in proximity) or without contact with cattle; confined breeding system (confined system: animals had no access to outside areas and were restricted to the bay) or semi-confined/extensive system (limited access or total access to pasture areas); activities of the horses (sport/exhibition; work/walk or recreation; reproduction); gender (male or female); age (≤ 2 years old or > 2 years old); racial definition (breeded horses or mixed-breed horses).

### DNA extraction

The extraction of deoxyribonucleic acid (DNA) from the blood samples was performed from 300 μL of blood using the Wizard® Genomic DNA Purification kit according to manufacturer’s recommendations.

For the DNA extraction from the tick samples, the specimens corresponding to each developmental stage were macerated in pools (nymphs = ten copies; larvae = ten copies; not engorged females = two copies; males = three copies), with the exception of the engorged females, which were macerated individually. After this step, genomic DNA was extracted based on the protocol for genomic DNA extraction according to Ferreira and Grattapaglia (1998). Each group of 11 samples had a control sample containing ultrapure water to rule out contamination during DNA extraction.

The DNA samples were resuspended in 200 μL of Tris–EDTA, quantified using the spectrophotometer Nanodrop ND-2000®, standardized at a concentration of 60 ng/μL and stored at −20 °C until use.

### Real-time PCR assay

The detection of *T*. *equi* DNA was performed using a TaqMan PCR system in a Real-Time StepOne Plus® instrument to amplify the 81-bp fragment of the *18S rRNA* gene. The assay used the Be18SF (5′-GCGGTGTTTCGGTGATTCATA-3′) and Be18SR (5′-TGATAGGTCAGAAACTTGAATGATACATC-3′) primer set and a fluorescent hydrolysis probe, Be18SP (5′-AAATTAGCGAATCGCATGGCTT-3′), which was labeled at the 5' end with the reporter dye 6-carboxyfluorescein and the 3' end with the quencher dye 6-carboxy-tetramethylrhodamine (Kim et al. 2008). The reactions were performed in duplicate on different plates with a final volume of 12 μL, which contained: 1X TaqMan® Universal PCR Master Mix, 450 nM of each primer, the 250 nM of hydrolysis probe and 180 ng of total DNA.

The thermocycling conditions were 50 °C for 2 min, 95 °C for 10 min, and 45 cycles at 95 °C for 20 s, followed by 55 °C for 1 min (Kim et al. 2008). The quantification cycle (*C*_q_) was standardized between plates and was manually allocated three cycles after the fluorescence base. Samples with *C*_q_ values less than or equal to 40 cycles were considered positive.

The analytical sensitivity of the assay was determined by evaluating the serial decimal dilutions of the *18S rRNA* gene of *T*. *equi* cloned into the plasmid pGEM-T® Easy Vector System. The sensitivity of the real-time PCR was evaluated with and without the addition of 1 μL of DNA from an uninfected blood sample and the addition of 1 μL DNA from an uninfected tick. The concentration and purity of the plasmid DNA were measured by spectrophotometer Nanodrop ND-2000®. The concentration of the plasmid DNA was the basis for calculating the plasmid number. For determining the analytical sensitivity of the qPCR, a curve was created using five points, which were plotted as the number of copies of plasmid based on *C*_q_ values. The number of copies ranged from 3 × 10^4^ to 3, and four separate dilutions were performed for each point on the curve.

### Statistical analysis

The horses’ positivity for *T*. *equi*, as determined by qPCR, was examined for association with epidemiological questionnaire variables using the chi-squared or Fisher’s exact tests at a 20 % significance level.

Among the independent variables that were statistically significant (*p* < 0.20) in the bivariate analysis, the Spearman correlation test was applied to check the collinearity and to eliminate highly correlated variables from the logistic regression model. In this test, the independent variables with *p* < 0.05 and *ρ* < 0.6 were included in the multiple logistic regression model. Among the two highly correlated variables (*p* < 0,05 and *ρ* ≥ 0,6), the one most biologically important was kept in the model.

A multiple logistic regression model was performed with the dependent variable (horses with molecular positivity for *T*. *equi*), which was dichotomous (negative = 0, positive = 1), as a function of the independent variables presented in the epidemiological questionnaire, at a 5 % level of significance.

In the tick samples, the presence of the infectious agent DNA was evaluated according to the species collected (using the genus, evolutionary stage, and the studied municipalities) by the chi-squared or Fisher’s exact tests at a 5 % significance level.

For the verification of the isolated effect of the presence of a tick variable on the presence of *T*. *equi* DNA in horses, a logistic regression model was established using a qualitative dichotomous variable (absence = 0 or presence = 1) for each tick species in relation to the presence of *T*. *equi* DNA in the horses. Based on these results, another simple logistic regression model was created to evaluate the level of *A*. *cajennense* infestation according to the presence of *T*. *equi* DNA in the animals. This tick infestation level was a qualitative and dichotomous variable (absent/low = 0 or moderate/high = 1) (Labruna et al. 2001).

All final logistic regression models were analyzed by a likelihood ratio. Statistical analyses were performed using the R programming package within the graphical interface R-Studio (Development Core Team 2010, http://www.r-project.org).

## Results

**Fig. 1:**
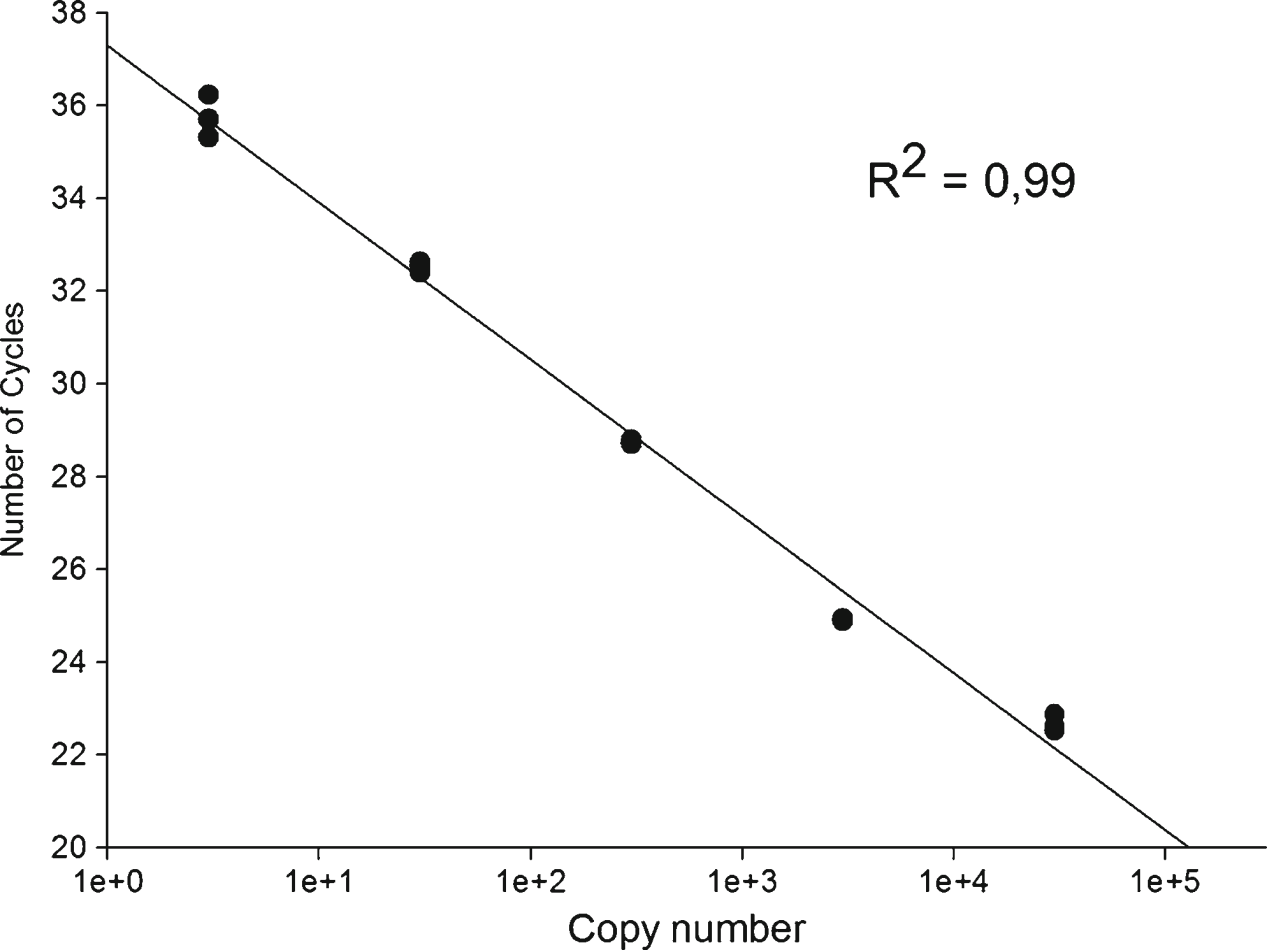
Standard curve plotted from serial decimal dilutions of plasmid DNA, containing the *Theileria equi 18S rRNA* gene (∼1,600 bp). The quantification cycle (*C*_q_) value obtained by real-time polymerase chain reaction using Taqman system was plotted as a function of the initial number of plasmid copies

**Fig. 2:**
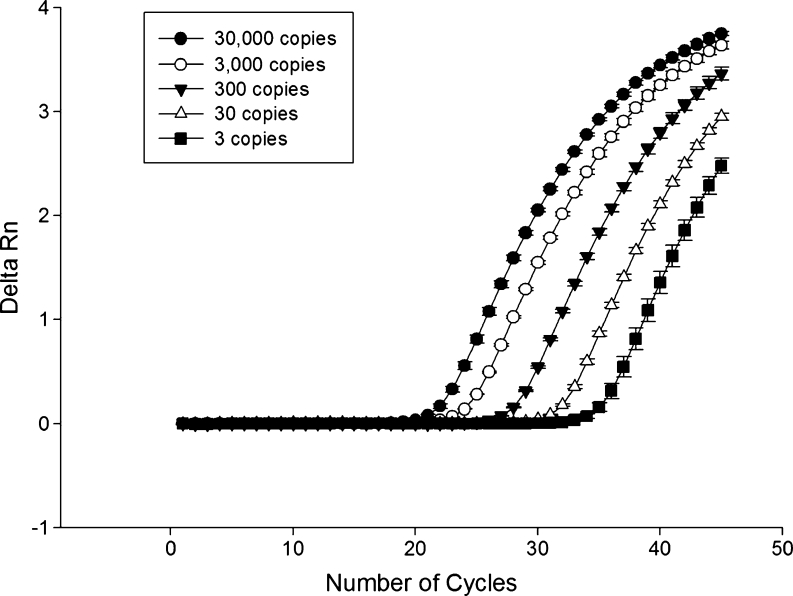
Real-time polymerase chain reaction analytical sensitivity, showing the amplification curves of plasmid DNA, containing the *Theileria equi 18S rRNA* gene (∼1,600 bp), in serial dilutions (3–30,000 copies)

The analytical sensitivity of the qPCR technique was evaluated using a known number of copies of the vector-insert. The detection limit of the technique was three copies of the plasmid containing the *18S rRNA* gene. The determination coefficient of four replications of five dilutions tested in the standard curve was 99 %, with varying *C*_q_ 22.64 ± 0.15 cycles in the first dilution (3 × 10^4^ copies of plasmid) to 35.73 ± 0.38 cycles in last dilution (three copies of the plasmid) (Fig. 1). No significant difference (*p* > 0.05) in analytical sensitivity when 1 μL of uninfected equine blood and tick DNA was added to the PCR. The curves show the same slope during the exponential phase of the amplification reaction demonstrating the efficiency of 97.65 %, even a point with few targets (Fig. 2).

Of the equine analyzed samples, 81 % (*n* = 253/314) exhibited fragmented amplification of the 81 bp of the *18S rRNA* gene specific for *T*. *equi* by qPCR. With regard to the analyzed cities, 91 % (*n* = 128/141) of the horses in Seropedica were considered positive for *T*. *equi*, significantly higher (*p* < 0.001) than the percentage of positive horses in Petropolis (72 %, *n* = 125/173) (Table 1).

**Table 1:** Bivariate and multivariate analysis of factors associated to *Theileria equi* infection in horses, by real-time PCR, according to animal characteristics, locality, zootechnical and sanitary management, contact with cattle, animals activity, breeding systems, ticks infestation in Petropolis and Seropedica municipalities, Rio de Janeiro, Brazil

Characteristic of the animals and management	Real-time PCR		Bivariate		Multivariate		
	Number of animals sampled	Positives (%)	*χ* ^2^	*P*	*P*	OR	CI 95 %
Gender							
Male	194	80.00	0.003	0.956	−	−	−
Female	120	80.93					
Age							
≤2 years	42	78.57	0.020	0.887	−	−	−
>2 years	272	80.88					
Racial definition							
Mixed breed	140	83.57	0.444	0.505	−	−	−
Defined breed	174	78.16					
Locality							
Petropolis (>400 m)	173	72.25	15.870	0.000	–^a^		
Seropedica (<400 m)	141	90.78			0.39	−	0.6–3.2
Zootechnical and sanitary management							
Satisfactory	150	66.67	33.803	0.000^b^	−	−	−
Unsatisfactory	164	93.29					
Contact with cattle							
No	218	77.06	4.900	0.027	–^a^		
Yes	96	88.54			0.48	−	0.2–1.8
Activity							
Sport/exposition	118	61.02	46.200	0.000	–^a^		
Work/walk	127	92.13			0.00	5.75	2.2–14.4
Reproduction	69	92.75			0.02	3.85	1.3–11.5
Breeding system							
Confined	19	78.95	−	0.970^c^	−	−	−
Extensive/semi-extensive	295	78.64					
Ticks infestation							
No	153	67.97	28.713	0.000	–^a^		
Yes	161	92.55			0.03	2.63	1.1–6.2
Animals’ origin							
Inside property	98	84.69	1.186	0.276	−	−	−
Outside property	216	78.70					

*χ* ^*2*^ value of chi-square test, *P p* value, *OR* odds ratio, *CI* confidence interval

^a^Category reference

^b^Removed from the multivariate analysis due to collinearity verified by Spearman test

^c^Fisher’s exact test

The variables inherent to equines, such as gender, racial definition, and age, were not associated with *T*. *equi* positivity (*p* > 0.20). However, with regard to variables related to ownership and breeding, municipality (*p* < 0.0001), property management (*p* < 0.00001), tick infestation of the animals (*p* < 0.0001), the horses in contact with cattle (*p* = 0.027), and the equine activity on-property (*p* < 0.0001) were associated with the presence of *T*. *equi* DNA in the samples (*p* < 0.20) (Table 1).

In the logistic regression model, only the equine activity on-property (*p* < 0.00001) and tick infestation (*p* = 0.02) were associated with *T*. *equi* infection. The property management variable (*p* < 0.00001) was removed from the multivariate analysis because it presented collinearity with the variable for the ticks infestation (*p* < 0.00001 and *ρ* = 0.6).

Compared with the horses that had activities related to sports or exhibition, the horses with activities related to work (draft animals in agriculture, riding animals for cattle handling) or walking (people transportation and recreation) were 5.75 times (CI, 2.3–14.4) more likely to be infected by *T*. *equi*. The horses with reproductive activity were 3.8 times (CI, 1.3–11.5) more likely to have the infection. Horses with an infestation of ticks were 2.6 times (CI, 1.1–6.2) more likely to present *T*. *equi* DNA.

Tick infestations were identified in 51.3 % (*n* = 161/314) of the evaluated horses. *A*. *cajennense* and *D*. *nitens* were found on these infected horses with frequencies of 47.8 % (*n* = 150/314) and 31.8 % (*n* = 100/314), respectively. Infestation with these two tick species was identified in 28.3 % (*n* = 89/314) of the evaluated horses. Among the 300 collected ticks, 63.7 % (*n* = 191/300) were identified as *A*. *cajennense* and 34.7 % (*n* = 104/300) as *D*. *nitens*. The ticks were primarily adults (*n* = 274/300, 91.3 %), followed by nymphs (*n* = 21/300, 7 %) and larvae (*n* = 5/300, 1.7 %). The specimens collected at an immature stage of development were identified taxonomically as larvae Ixodida. In relation to the ticks’ gender, 61 % (*n* = 183/300) were identified as females and 30.3 % (*n* = 91/300) as males.

Regarding the detection of *T*. *equi* DNA in the evaluated ticks, 7.7 % (*n* = 23/300) of the ticks were positive. Furthermore, 9.9 % (*n* = 19/191) of the *A*. *cajennense* and 3.8 % of the *D*. *nitens* (*n* = 4/104) had amplified the 81-bp fragment from the *T*. *equi 18S rRNA* gene, with no statistically significant difference (*p* = 0.07) between the species (Table 2). Nevertheless, it is noteworthy that although not significantly different in frequency, the *A*. *cajennense* samples were 2.6 times more likely to be considered positive for *T*. *equi* than the *D*. *nitens* samples. The *T*. *equi* DNA fragment was not detected in the nymphs (*n* = 0/21) or larvae (*n* = 0/5) samples. The males were significantly more likely (*p* < 0.05) to be positive for *T*. *equi* (15.4 %, *n* = 14/91) than the females (4.9 %, *n* = 9/183). The males were more likely (*p* < 0.05) to be positive for *T*. *equi* (18.2 %, *n* = 12/66) than the *A*. *cajennense* females (5.8 %, *n* = 7/121). However, the *D*. *nitens* males (8 %, *n* = 2/25) and females (3.2 %, *n* = 2/62) did not significantly differ (*p* > 0.05) in their positivity for *T*. *equi* (Table 2).

**Table 2:** Bivariate analysis of factors associated to *Theileria equi* infection in tick samples, by real-time PCR, according to samples characteristics, as locality, identified species, developmental stage, and tick gender in the state of Rio de Janeiro, Brazil

Tick samples characteristics	Real-time PCR		Bivariate	
	Number of animals sampled	Positives (%)	*χ* ^2^	*P*
Altitude				
<400 m (Seropedica)	238	6.69	−	0.28^a^
>400 m (Petropolis)	62	11.29		
Species				
* Amblyomma cajennense*	191	9.95	3.96	0.07^a^
* Dermacentor nitens*	104	3.85		
Developmental stage				
Adults	274	8.39	2.36	0.31
Nymphs	21	0.00		
Larvae	5	0.00		
Gender				
Male	91	15.38	11.77	0.00
Female	183	4.92		
*Amblyomma cajennense*				
Male	66	18.18	7.78	0.02
Female	121	5.79		
Nymphs	4	0.00		
*Dermacentor nitens*				
Male	25	8.00	1.91	0.38
Female	62	3.23		
Nymphs	17	0.00		

*χ* ^*2*^ value of chi-square test, *P p* value

^a^Fisher’s exact test

The logistic regression model, which was applied to the tick species in relation to the presence of *T*. *equi* DNA in the horses, indicated that the horses infested with *A*. *cajennense* were four times more likely to harbor *T*. *equi* (Table 3). Using a simple logistic regression model to evaluate the *A*. *cajennense* infestation level in comparison with the animals’ positivity, the horses with moderate to high infestation were 2.7 times more likely to have *T*. *equi* DNA than the horses with low-level or no *A*. *cajennense* infestation (Table 4).

**Table 3:** Multivariate analysis of *Theileria equi* positivity in horses by real-time PCR, in relation to the tick species observed on animals in the state of Rio de Janeiro, Brazil

Ticks	Real-time PCR		Multivariate		
	Number of animals sampled	Positives (%)	*P*	OR	CI
*Amblyomma cajennense*					
Yes	150	92.7	0.00	4.10	(1.8–9.1)
No	164	69.5	–^a^	−	−
*Dermacentor nitens*					
Yes	100	93.0	0.18	−	−
No	214	74.8	–^a^	−	−

*P p* value, *OR* odds ratio, *CI* confidence interval

^a^Category reference

**Table 4:** Simple logistic regression model to evaluate the *Amblyomma cajennense* infestation level in comparison with the animals´ positivity for *Theileria equi* in the state of Rio de Janeiro, Brazil

*Amblyomma cajennense*	Real-time PCR		Logistic regression		
	Number of animals sampled	Positives (%)	*P*	OR	CI
Infestation level					
Moderate/many	74	90.5	0.01	2.77	(1.2–6.4)
None/few	240	77.5	–^a^	−	−

*P p* value, *OR* odds ratio, *CI* confidence interval

^a^Category reference

## Discussion

This epidemiological study reinforces the endemic character that equine theileriosis assumes in Brazil (Pfeifer-Barbosa et al. 1995; Heuchert et al. 1999; Golynski et al. 2008; Kerber et al. 2009; Santos et al. 2011). In addition, this study highlights the possible factors relevant to properties, breed management, and animal characteristics that are associated with *T*. *equi* presence in horses. It also demonstrates the importance of epidemiology as a vector research tool to understand the possible role of other tick species in addition to *R*. *microplus* in the transmission and spread of *T*. *equi* in Brazil.

The high frequency of *T*. *equi* among the horses in the studied municipalities is in agreement with several serological studies conducted in areas located near the studied municipalities (Pfeifer-Barbosa et al. 1995; Santos et al. 2011). However, importantly, this study detected *T*. *equi* DNA using qPCR, a highly sensitive and specific technique used in many molecular epidemiology investigations (Monis et al. 2005).

The municipality of Seropedica, which is located in the low-altitude region of the state of Rio de Janeiro, has a semi-humid tropical climate, with an annual average temperature of approximately 24 °C. In contrast, the municipality of Petropolis, which is located in the high-altitude region of the state, has a tropical climate and an annual average temperature of approximately 16 °C, with winter temperatures that may reach 0 °C. The conditions encountered by tick vectors in high-altitude cities such as Petropolis are less favorable for their development and reproduction, which contributed to the lower tick infestation and the lower frequency of *T*. *equi* infection in the horses in that location (Davey and Cooksey 1989; Chacón et al. 2003). Furthermore, management conditions and the quality of the properties found in Petropolis are better than those of properties in Seropedica.

According to the bivariate analysis, the *T*. *equi* positivity of the horses bred in close contact with cattle was significantly different from the *T*. *equi* positivity of the horses bred in the absence of cattle. This observation suggests a possible involvement of *R*. *microplus* on the epidemiological chain of equine theileriosis in the studied region. Experimental studies indicate that *R*. *microplus* is capable of being infected and is capable of transmitting the equine theileriosis agent when feeding on horses chronically infected or with an acute infection of *T*. *equi* (Guimarães et al. 1998a, b; Stiller et al. 2002; Ueti et al. 2008). Furthermore, horses with an infestation of *R*. *microplus* have been observed in areas where cattle graze (Bittencourt 1990; Labruna et al. 2001). However, it is unlikely that *R*. *microplus* plays an important role in the equine theileriosis enzootic cycle in the studied region because this study found no horses infested by this tick species. Perhaps other forms of transmission or other arthropods may be involved in maintaining the high level of the disease observed in the studied area.

The horses activities related to work, walking, and reproduction were factors that determined the infection with *T*. *equi*. This finding was observed by Abutarbush et al. (2012), in which the animals used for work showed a higher frequency of *T*. *equi* positivity, which differed significantly from other variables, such as the animals raised for practicing polo, running, jumping, reproduction, and exposure. These findings may also be related to the activities that the horse exerts on the property, where in many cases, the animals with work or walking activities also move into large pasture perimeters and increase their chances of infestation by potential tick vectors. Furthermore, animals with work, walking, and reproduction activities often live on properties that have poor management (Santos et al. 2011; Heuchert et al. 1999), are in close contact with cattle in pastures infested by different tick species (Heuchert et al. 1999), and have a grazing habit (Abutarbush et al. 2012; Nizoli et al. 2008), so they are at increased risk of contact with tick vectors. In our study, we observed that animals destined for sports and exposure activities receive management considered ideal for these types of breeding, with appropriate zootechnical buildings where the animals receive balanced meals, systematic veterinary care, and sanitary control; thus, they are at decreased risk of tick vector infestation.

The experimental transmission of *T*. *equi* is associated with *Rhipicephalus* ticks (Guimarães et al. 1998a, b; Stiller and Coan 1995; De Waal 1992; Ueti et al. 2005). However, to date, there are no reports that demonstrate the truly natural vector of this etiologic agent of importance in horses worldwide. Studies suggest that the species *A*. *cajennense* can act as a natural vector (Scoles et al. 2011; Kerber et al. 2009) of this agent because of its increased dispersion and predilection for horses. Nevertheless, in an experimental study conducted by Ribeiro et al. (2011), *T*. *equi* infection was not observed in *A*. *cajennense* that fed on horses with an acute or chronic infection. This study demonstrated that *A*. *cajennense* is associated with the presence of *T*. *equi* DNA in horses, but the participation of *A*. *cajennense* as a natural vector remains unclear. Thus, the high frequency of *A*. *cajennense* infestation among the horses may be considered a confounding factor in the epidemiological analysis. In support of this confounding factor are studies demonstrating that *T*. *equi* can be transmitted congenitally in equines (Santos et al. 2008), making vector investigation through epidemiological analysis difficult. However, in a study conducted in Texas in the USA, Scoles et al. (2011) found that *A*. *cajennense* was capable of becoming naturally infected by *T*. *equi* and was able to experimentally transmit it when feeding on infection-free horses, demonstrating that *T*. *equi* can be transmitted in an interstadial way by this tick species.

In this study, *A*. *cajennense* males were significantly more likely than females to have *T*. *equi* DNA. The *A*. *cajennense* males are able to feed on various hosts during their lifecycle, increasing their chances of becoming infected with *T*. *equi*, and they survive for long fasting periods (Serra-Freire 1982; Oliveira et al. 2003). These characteristics make them important transmitters of etiologic agents in animals and humans.

The high frequency of *T*. *equi* in horses in Brazil and around the world suggests that the natural vector is abundant among the animals, is common in all locations where horse breeding occurs, and is capable of transmitting *T*. *equi* from an infected animal to a susceptible one. Therefore, epidemiologically, *A*. *cajennense* could be a suitable vector in the studied area because it requires three hosts to complete its life cycle, is distributed throughout the Americas, and seems to prefer horses as the hosts for all parasitic stages (Aragão 1936; Oliveira et al. 2000). In this study, the horses infested with *A*. *cajennense* at moderate or high levels were more likely to be infected with *T*. *equi*, corroborating previous findings.

The qPCR technique was an excellent tool for epidemiological hemoparasite investigation because *T*. *equi* has a chronic nature in the equine organism. Infestation by ticks and animal activities are factors associated with *T*. *equi* infection in the studied region. Among the investigated ticks, *A*. *cajennense* was the species epidemiologically regarded as a potential biological vector of this piroplasma under natural conditions in the studied area.
